# An Engineered Pathway for Production of Terminally Sialylated *N*-glycoproteins in the Periplasm of *Escherichia coli*

**DOI:** 10.3389/fbioe.2020.00313

**Published:** 2020-04-15

**Authors:** Jing Zhu, Yao Ruan, Xin Fu, Lichao Zhang, Gaoshun Ge, J. Gerard Wall, Teng Zou, Yang Zheng, Ning Ding, Xuejun Hu

**Affiliations:** ^1^Academic Centre for Medical Research, Medical College, Dalian University, Dalian, China; ^2^Centre for Research in Medical Devices (CÚRAM) and Microbiology, School of Natural Sciences, National University of Ireland, Galway, Ireland

**Keywords:** sialylation, sialic acid, *Escherichia coli*, *N*-glycoprotein, α-2, 6-sialyltransfease

## Abstract

Terminally sialylated *N*-glycoproteins are of great interest in therapeutic applications. Due to the inability of prokaryotes to carry out this post-translational modification, they are currently predominantly produced in eukaryotic host cells. In this study, we report a synthetic pathway to produce a terminally sialylated *N*-glycoprotein in the periplasm of *Escherichia coli*, mimicking the sialylated moiety (Neu5Ac-α-2,6-Gal-β-1,4-GlcNAc-) of human glycans. A sialylated pentasaccharide, Neu5Ac-α-2,6-Gal-β-1,4-GlcNAc-β-1,3-Gal-β-1,3-GlcNAc-, was synthesized through the activity of co-expressed glycosyltransferases LsgCDEF from *Haemophilus influenzae*, *Campylobacter jejuni* NeuBCA enzymes, and *Photobacterium leiognathi* α-2,6-sialyltransferase in an engineered *E. coli* strain which produces CMP-Neu5Ac. *C. jejuni* oligosaccharyltransferase PglB was used to transfer the terminally sialylated glycan onto a glyco-recognition sequence in the tenth type III cell adhesion module of human fibronectin. Sialylation of the target protein was confirmed by lectin blotting and mass spectrometry. This proof-of-concept study demonstrates the successful production of terminally sialylated, homogeneous *N*-glycoproteins with α-2,6-linkages in the periplasm of *E. coli* and will facilitate the construction of *E. coli* strains capable of producing terminally sialylated *N*-glycoproteins in high yield.

## Introduction

*Escherichia coli* is a commonly used host for the production of biotherapeutic and other high-value proteins. A favored expression strategy is to export the protein of interest to the periplasm to simplify downstream processing and facilitate disulfide bond formation (Karyolaimos et al., 2019; Tripathi and Shrivastava, 2019). Yields of several g/L of active human growth hormone, containing two disulfide bonds and exported to the periplasm using the Tat pathway, were recently demonstrated in *E. coli “*TatExpress” strains (Guerrero et al., 2019). The lack of a natural pathway to achieve *N*-glycosylation remains a major limitation of *E. coli* as an expression host, however. Furthermore, as *E. coli* does not naturally produce sialic acids, it remains a particular challenge to produce terminally sialylated *N*-glycans, characteristic of many human *N*-glycoproteins, in the organism.

Terminal sialic acid has a key impact on the properties of *N*-glycoproteins. Due to its strong electronegativity, sialic acids can increase the solubility or resistance to proteolytic degradation of a glycoprotein, as well as enhancing its residence time in blood and promoting transportation of drugs and ions into cells (Aquino et al., 1980; Raju et al., 2001; Bork et al., 2009; Meuris et al., 2014; Cuccui and Wren, 2015; Gupta and Shukla, 2018; Thi Sam et al., 2018). It is unsurprising, therefore, that extensive research efforts have focused on overcoming the traditional bottlenecks associated with recombinant production of terminally sialylated glycoproteins of biopharmaceutical importance.

Sialylated therapeutic *N*-glycoproteins are predominantly produced in eukaryotic expression systems such as plants (Montero-Morales et al., 2019) and mammalian cell lines, particularly Chinese hamster ovary (CHO) cells and human embryonic kidney (HEK) cells, even though these platforms are associated with incomplete glycosylation and low efficiency of sialylation (Thi Sam et al., 2018). Engineering approaches to resolve these limitations have included simultaneous overexpression of human beta-galactoside α-2,6 sialyltransferase I and UDP-GlcNAc 2-epimerase/ManNAc kinase, resulting in yields of sialylated *N*-glycoprotein increased more than 7-fold compared to the wild-type (Thi Sam et al., 2018). Yeasts have also been successfully engineered to produce humanized *N*-glycoproteins modified with terminally sialylated biantennary glycans [Sia2Gal2GlcNAc2Man3GlcNAc (90.5%) and SiaGal2GlcNAc2Man3GlcNAc (7.9%)] (Hamilton et al., 2006), though neither of these approaches yielded sialylated, homogeneous *N*-glycoproteins. Meuris and co-workers developed HEK 293S-derived GlycoDelete cells with a shortened Golgi *N*-glycosylation pathway to produce terminally sialylated *N*-glycoproteins (Meuris et al., 2014), leading to approximately 75% of produced proteins containing a terminally sialylated *N*-glycan trisaccharide (Neu5Ac-α-2,3-Gal-β-1,4-GlcNAc-). Proteins modified with the sialylated trisaccharide exhibited reduced initial serum clearance and did not acquire new immunogenic epitopes.

Bacterial *N*-linked glycosylation systems have also been explored as a means of producing sialylated *N*-glycoproteins due to the constraints of long time and high costs associated with producing heterologous proteins in mammalian cells. The successful functional transfer of the *N*-glycosylation pathway from *Campylobacter jejuni* to *E. coli* in 2002 raised the possibility of producing *N*-glycoproteins with customized glycans in the *E. coli* periplasm (Wacker et al., 2002). Since then, some successes have been reported in engineering *N*-glycoproteins with more human-like glycan motifs than the original *C. jejuni* pattern, such as the Lewis × (Le^x^) glycan epitope and eukaryotic Man3GlcNAc2 core glycan (Hug et al., 2011; Valderrama-Rincon et al., 2012). Sialylation is critical to enhancing the circulatory residence time of glycoproteins (Meuris et al., 2014), however, and the reported systems would require multiple additional engineering steps to generate humanized terminally sialylated *N*-glycoproteins (Cuccui and Wren, 2015).

The characterization of an *N*-linking glycosyltransferase (NGTase) in the respiratory swine pathogen *Actinobacillus pleuropneumoniae* has provided an alternative approach to produce *N*-glycoproteins in *E. coli*. Keys and co-workers reported a biosynthetic pathway, based on NGTase, for site-specific polysialylation of recombinant proteins with α-2,8-linked polysialic acid (polySia) chains in the *E. coli* cytoplasm, albeit with only approximately 20% of target molecules modified with polySia (Keys et al., 2017). Other workers have achieved approximately 62% glycosylation of *N*-glycoproteins with an α-2,3-linked, terminally sialylated *N*-glycan trisaccharide (Neu5Ac-α-2,3-Gal-β-1,4-GlcNAc-) (Tytgat et al., 2019). Scale-up of either approach is limited by the need to supply the expensive and unstable donor Neu5Ac, however. Meanwhile, no terminally sialylated homogeneous *N*-glycoproteins with α-2,6-linkages have yet been produced in the periplasm of *E. coli*.

α-2,3, α-2,6, and α-2,8 linkages have all been observed in terminally sialylated human *N*-glycoproteins, with Neu5Ac the most abundant sialic acid. α-2,6-linked sialic acid predominates in human immunoglobulins and is critical for their anti-inflammatory activity (Jefferis, 2006; Kaneko et al., 2006; Anthony et al., 2008; Anthony and Ravetch, 2010), unlike α-2,3-linked sialic acid residues which exhibit no such activity (Anthony and Ravetch, 2010). A mechanism to produce homogeneous, terminally sialylated *N*-glycoproteins with α-2,6-linkages is highly desired, therefore, as a step toward reducing the immunogenicity and increasing the serum half-life of biomedically important glycoproteins.

In this work, we describe the production of a terminally sialylated homogeneous *N*-glycoprotein in the periplasm of an engineered *E. coli* host. The *E. coli* DH5α strain was engineered to synthesize CMP-Neu5Ac and assemble a terminally sialylated glycan on an undecaprenyl-pyrophosphate lipid carrier by combining biosynthetic pathways for CMP-Neu5Ac and a tetrasaccharide human glycan mimic. Sialylation was achieved using α-2,6-STase from *Photobacterium leiognathi* JT-SHIZ-145 (*pl-ST6*) (Yamamoto et al., 2007), and the sialylated glycan was transferred on a glyco-tagged acceptor protein by OTase PglB from *C. jejuni*. This approach offers a pathway to economical production of terminally sialylated homogeneous *N*-glycoproteins.

## Materials and Methods

### Plasmids and Biochemical Pathway Construction

The plasmids used in this study are listed in Table 1 and full sequences are available under the associated GenBank accession numbers. Plasmid maps are provided in Supplementary Figure 1. Enzymes for synthesis of the sialylated glycan included (i) *Haemophilus influenzae* LsgCDEF glycosyltransferases (GTases) to produce lipid-linked oligosaccharides (LLOs), which consisted of tetrasaccharide glycan Gal-β-1,4-GlcNAc-β-1,3-Gal-β-1,3-GlcNAc- (Mandrell et al., 1992; Phillips et al., 2000), (ii) *C. jejuni* NeuBCA synthetases for synthesis of Neu5Ac and CMP-Neu5Ac, utilizing UDP-GlcNAc as the starting substrate *in vivo* (Drouillard et al., 2010), and (iii) *P. leiognathi* α-2,6-STase *pl-ST6* for sialylation (Yamamoto et al., 2007), with *C. jejuni* OTase PglB achieving transfer of the end glycan to the glyco-tagged recombinant proteins. *LsgCDEF* and *PglB* genes were amplified by PCR from pGEMLOS-5 and pACYCpgl plasmids, respectively. Other genes used in the study were synthesized by Convenience Biology Corporation (Changzhou, China) and GenScript (Nanjing, China) with parameters optimized by OptimumGene^TM^ software for expression in *E. coli*. The human tenth fibronectin type III domain (*FN3*) gene, *pl-ST6*, and *neuBCA* were cloned into pIG6 to generate plasmid pIG6-Sia containing a *lac* promoter *– ompA* leader peptide*- FLAG – FN3 – DQNAT* glycosylation site *–* 6xHis *–* T7 terminator *–* P regulatory region *– rbs – pl-ST6 – ompA rbs – neuBCA* expression cassette. The P regulatory region occurs upstream of the *N*-glycosylation *pgl* locus in *C. jejuni*. *pglB*, *lsgCDEF*, GlcNAc-phosphate transferase gene *wecA* and membrane translocase gene *pglK* were cloned in the pIG6-compatible plasmid pACYC184 to generate plasmid pC15-plsg with the following format: Arabinose promoter *– rbs – pglB – rbs1* – *wecA – rbs2- pglK – ompA rbs – lsgCDEF – rrnB* terminator. The expression plasmid pIG6-FN3-Gly-1 was previously described for the production of FN3 and glycosylated FN3 proteins (Ding et al., 2017). The sequences of all plasmids were confirmed by DNA sequencing.

**TABLE 1:** Key strains, genes and plasmids used in this study.

			**GenBank accession No.**
**Strain/gene/plasmid**	**Genotype or description**	**Reference or source**	**or Gene ID No.**
**Strains**			
JM109	*recA1, endA1, gyrA96, thi-1, hsdR17(rk^–^mk^+^), e14^–^(mcrA^–^)supE44, relA1*, Δ(*lac-proA*B)/F′[*traD36, proAB^+^, l-acI^q^, lacZ*ΔM15]	Takara Biotechnology (Dalian) Co., LTD	
DH5α	F*^–^*, φ 80d/*lacZ* ΔM15, Δ(*lacZYA-argF*)U169, *deoR*, *recA1*, *endA1*, *hsdR17* (*rK^–^, mK^+^*), *phoA*, *supE44*, λ*^–^*, *thi-1*, *gyrA96*, *relA1*	Takara Biotechnology (Dalian) Co., LTD	
DKK601	DH5αΔ*nanKETA*::*kan*, Kan^R^	This study	
**Genes**			
*glyco-tagged FN3*	glyco-tagged human tenth fibronectin type III domain gene	This study	MK355444
*wecA*	GlcNAc-phosphate transferase from *Escherichia coli*		948789
*lsgCDEF*	glycosyltransferases for synthesis of the glycan (Gal-β-1,4-GlcNAc-β-1,3-Gal-β-1,3-GlcNAc-) from *Haemophilus influenzae*		M94855.1
*pglK*	membrane translocase gene from *Campylobacter jejuni*		905421
*pglB*	OSTase gene from *Campylobacter jejuni*		905417
*neuBCA*	synthetase genes for synthesis of Neu5Ac and CMP-Neu5Ac from *Campylobacter jejuni*		AF400048.1
*pl-ST6*	2,6-sialyltransferase gene from *Photobacterium leiognathi JT-SHIZ-145*		AB306315
**Plasmids**			
pACYCpgl	carrying the *N*-glycosylation *pgl* locus from *Campylobacter jejuni*, Cm^R^	Feldman et al., 2005	
pACYC184	P15A ori, Cm^R^		X06403.1
pIG6	expression vector, *lac* promoter, *ompA* leader peptide, target protein into periplasm, high-copy, Amp^R^	Ge et al., 1995	
pGEMLOS-5	carrying *lsgCDEF* cluster from *Haemophilus influenzae*, Amp^R^	Phillips et al., 2000	
pIG6-FN3-Gly-1	glyco-tagged FN3 (MK355444) expression vector, includes *lac* promoter – *ompA* leader peptide – *FN3* – DQNAT sequon – 6xHis – Terminator, Amp^R^; vector backbone is pIG6	Ding et al., 2017	
pC15-plsg	“*N*-glycosylation pathway,” includes rbs – *pglB* – rbs1 – *wecA* – *wzzE* rbs2 – *pglK* – *ompA* rbs – *lsgCDEF* – *rrnB* terminator, all under control of an Arabinose promoter, Cm^R^; vector backbone is pC15. rbs1 and rbs2 both were from artificial synthesis sequences based on the rbs (core sequence: AGGA) of pET28a (Novagen)	This study	MK353498
pIG6-Sia	“sialylation pathway,” includes *Lac* promoter – *ompA* leader peptide – FN3 – DQNAT sequon – T7 terminator – P regulatory region – *pl-ST6* – *ompA* rbs – *neuBCA*, Amp^R^; vector backbone is pIG6. P regulatory region: the upstream sequence of the *N*-glycosylation *pgl* locus from *Campylobacter jejuni*	This study	MN721873

### Production and Purification of Sialylated Proteins

Bacterial strains used are listed in Table 1. Primers used in this study are listed in Supplementary Table 1. *E. coli* JM109 was used for maintenance and propagation of plasmid DNA. To facilitate *in vivo* production of CMP-Neu5Ac, a *nanKETA* cluster knock out was created in *E. coli* K12-derivative DH5α by λ Red homologous recombination (Fierfort and Samain, 2008). The resultant DH5αΔ*nanKETA*:*kan* strain which was confirmed by sequencing, termed DKK601, lacks β-galactosidase activity which could cleave *N*-linked lactose produced during glycosylation. For production of terminally sialylated *N*-glycoprotein, a single, freshly transformed colony of *E. coli* DKK601 containing pIG6-Sia and pC15-plsg plasmids was inoculated from a Luria-Bertani (LB) agar plate containing 100 μg ml^–1^ ampicillin and 70 μg ml^–1^ chloramphenicol into 5 ml LB with the same antibiotics and grown overnight with shaking at 37°C. This culture was used to inoculate 500 ml LB containing the same antibiotics, followed by shaking at 28°C and the addition of 200 μg ml^–1^ L-arabinose and 1 mM isopropyl β-D-thiogalactoside (IPTG) when the OD_600_ reached 0.6. Expression was allowed to continue for 5 h. Bacterial growth rate was monitored by recording the optical density of the culture at 600 nm over time using a spectrophotometer. The expression and sialylation of target proteins was monitored by western blot from 0 to 5 h induction. After harvesting of cells by centrifugation, FN3 acceptor proteins were purified using 1 ml HisTrap columns, and desalted using a PD-10 column (Hu et al., 2013). Unglycosylated FN3 and glycosylated FN3 with the tetrasaccharide glycan (Gal-β-1,4-GlcNAc-β -1,3-Gal-β-1,3-GlcNAc-) were produced and purified as previously reported (Ding et al., 2017). Protein concentrations were determined by BCA assay (Pierce).

### Immunoblot Analysis

Purified FN3 proteins (sialylated, un-sialylated and unglycosylated formats) were separated on 15% SDS-PAGE gels and analyzed by Coomassie Blue staining and immunodetection using a monoclonal anti-FLAG M1 antibody (Sigma-Aldrich). HRP-conjugated rabbit anti-mouse IgG (Invitrogen) was used as the secondary antibody. Proteins of varying glycosylation status were also detected with ECA-HRP and SNA-I-HRP lectins (EY Laboratories). Blots were visualized using a Chemidoc^TM^ XRS + system (Bio-Rad) and analyzed by Image Lab software. The data are reported from at least three replicate experiments.

### LC-MS/MS Analysis

Approximately 50 μg of purified protein was loaded onto 7-kD Spin Desalting Columns and eluted with 50 mM ammonium bicarbonate (Sigma-Aldrich, pH 7.5) in a 20 μl volume, followed by incubation at 56°C for 1 h. Samples were cooled to room temperature and digested with l μg of trypsin at 37°C for 14 h, followed by inactivation of trypsin at 80°C for 10 min. After cooling to room temperature, l μg of Glu-C endoproteinase was added and samples were incubated at 37°C for 14 h, and dried. Following re-solubilization with 0.1% TFA, samples were quantified using a Nanodrop 2000 and 500 ng of peptides with glycans were analyzed by Thermo Orbitrap Exactive HF mass spectrometer (Thermo Fisher). The mobile phase comprised 0.1% formic acid in water (eluent A) and 0.1% formic acid in 99.9% acetonitrile solution (eluent B). Elution was at a flow rate of 0.6 μl min^–1^ using three linear gradients steps: from 6 to 30% acetonitrile in 34 min, from 30 to 40% acetonitrile in 7 min, and from 40 to 95% acetonitrile in 4 min, with constant 0.1% formic acid. For exact mass measurement with a lock spray, the capillary voltage was set at 2.2 kV, the temperature at 320°C and the normalized collision energy at 50%. The AGC target of full scan MS (200–2500 *m/z*) was 3 × 10^6^ and data acquisition was in the Q-Orbitrap at a resolution of 120,000.

### Database Analysis and Identification of Modified Residues

Spectra of the digested glycopeptides were searched with the Byonic software, which is defined by the absolute quality of the peptide-spectrum match over 300. Both the full MS and MS/MS scans were with a tolerance of 15 ppm. The presence of oxonium ions for NeuAc (292.10) and NeuAc-H_2_O (274.09) in MS/MS spectra were used to scout for sialylated glycopeptides. Indexed databases for semi-cut trypsin and Glu-C digests were created, allowing for up to three missed cleavages, and the sequencing of the peptide was performed manually. Assignments of all spectra in samples were validated by manual inspection for the precursor isotope pattern and expected glycan fragments.

## Results

### Design of the Biosynthetic Pathway

**FIGURE 1:**
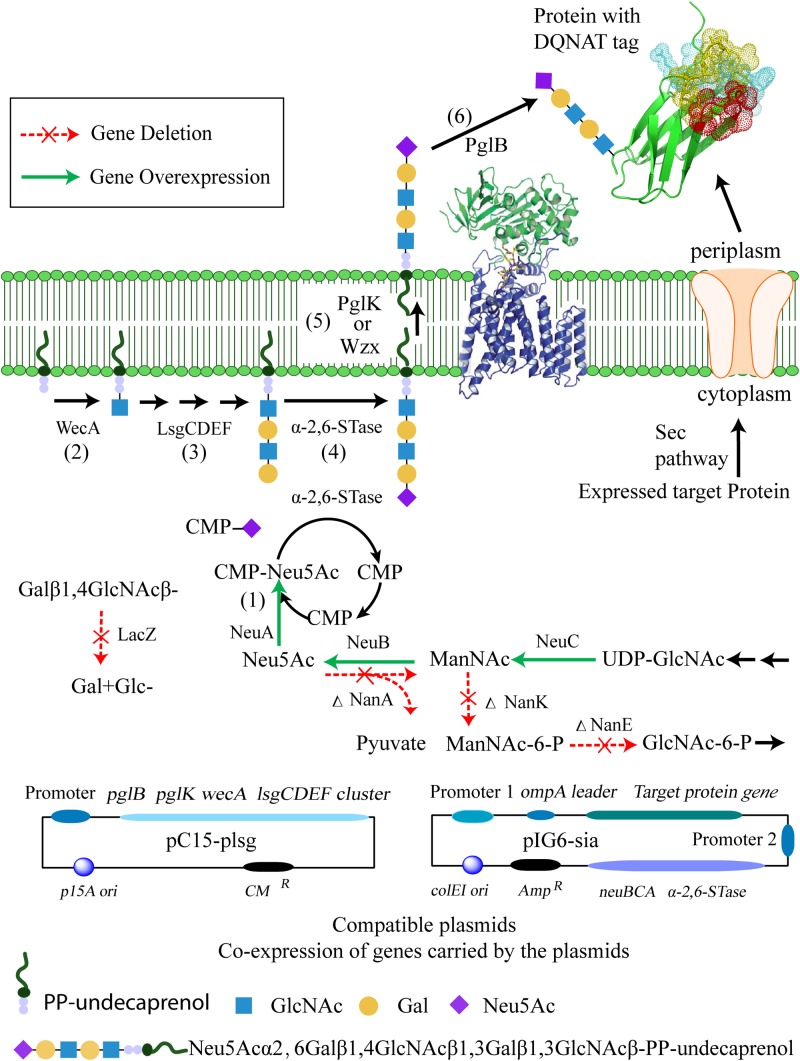
The proposed biosynthetic pathway for the production of terminally sialylated proteins in the periplasm of *E. coli*, and associated plasmid constructs. Synthesis of CMP-Neu5Ac is achieved by the sub-pathway constructed with overexpressed *neuBCA* genes from *C. jejuni* (1). Synthesis of the pentasaccharide LLOs Gal-β-1,4-GlcNAc-β-1,3-Gal-β-1,3-GlcNAc-β-pp-undecaprenol and Neu5Ac-α-2,6-Gal-β-1,4-GlcNAc-β -1,3-Gal-β-1,3-GlcNAc- is achieved by the sub-pathway constructed with GlcNAc-1-phosphate glycosyltransferase (WecA) enzyme (2), glycosyltransferases (LsgCDEF) (3), and the α-2,6-STase (*pl-ST6*) enzyme (4). The sialylated glycan is flipped by PglK (or Wzx) flipases from the cytoplasmic to the periplasmic side of the membrane (5). The final modification of the target protein with the synthesized glycan is achieved by PglB (6).

In this work, we designed a pathway to produce a terminally sialylated, homogeneous *N*-glycoprotein with a short glycan in the *E. coli* periplasm (Figure 1). The approach consists of two main independent sub-pathways: synthesis of CMP-Neu5Ac and the tetrasaccharide glycan Gal-β-1,4-GlcNAc-β-1,3-Gal-β-1,3-GlcNAc-. The pathway involves six steps: (1) To provide CMP-Neu5Ac for sialylation, the CMP-Neu5Ac is synthesized by NeuBCA synthases, utilizing UDP-GlcNAc as substrate; (2) the precursor of lipid-linked oligosaccharides (LLOs) is synthesized by WecA to provide the starting sugar; (3) which is then extended by LsgCDEF GTases at the inner surface of cytoplasmic membrane to synthesize the tetrasaccharide glycan Gal-β-1,4-GlcNAc-β-1,3-Gal- β-1,3-GlcNAc-; (4) followed by transfer of Neu5Ac onto this tetrasaccharide glycan via an α-2,6-linkage by the use of the *pl-ST6*α-2,6-STase; (5) flipping of the sialylated glycan from the cytoplasmic to the periplasmic side of the membrane by PglK (or Wzx) flippases; (6) and, finally, the sialylated glycan is transferred to the glyco-tagged (DQNAT motif) (Lizak et al., 2011) recombinant protein using OTase PglB from *C. jejuni*.

### Production of Terminally Sialylated, Homogeneous *N*-glycosylated FN3 in the *E. coli* Periplasm

To explore the production of a terminally sialylated homogeneous *N*-glycoprotein, we utilized FN3 with an N-terminal *ompA* leader peptide, FLAG and hexahistidine tags and a single engineered glycosylation site (DQNAT motif) (Lizak et al., 2011) as an acceptor protein. The FN3 was expressed and exported to the periplasm through the Sec pathway. To date, monobodies based on FN3 have been generated with nanomolar and picomolar range affinities for use in a number of emerging therapeutic applications (Sullivan et al., 2013).

**FIGURE 2:**
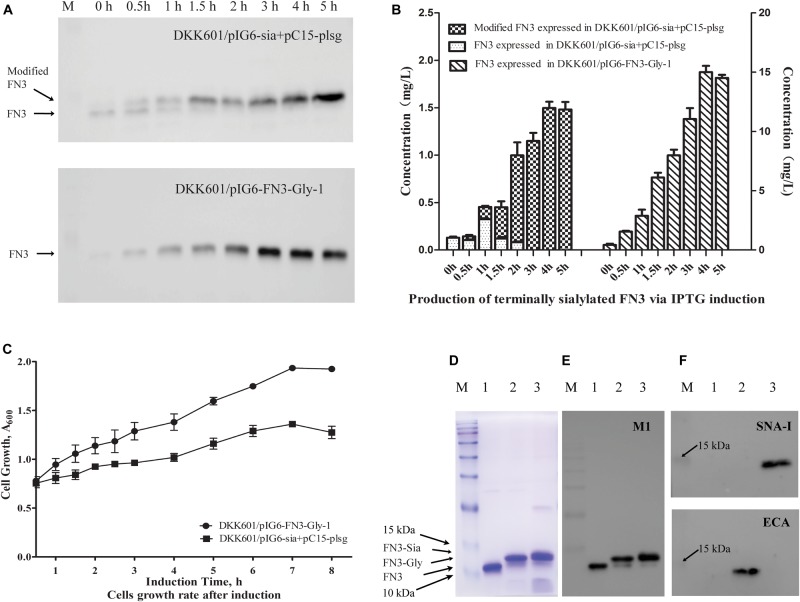
Production of terminally sialylated homogeneous *N*-glycosylated FN3 in the *E. coli* periplasm. **(A)** Western-blot analysis of the expression of modified FN3. *E. coli* cell lysates containing pIG6-Sia + pC15-plsg (upper panel) or pIG6-FN3-Gly-1 plasmids (lower panel) were detected at the indicated induction time points using anti-FLAG M1 antibody, with quantification of protein yields at different induction time points to determine optimal expression conditions (For detailed information, see Supplementary Figures 3, 4) **(B)**. **(C)** Growth curves of *E. coli* DKK601/pIG6-FN3-Gly-1 (expressing FN3 protein without sialylation pathway) and *E. coli* DKK601/pIG6-Sia + pC15-plsg (expressing modified FN3 protein with sialylation pathway) cells after induction of recombinant protein expression and glycosylation. Purified unmodified FN3, *N*-glycosylated FN3 (FN3-Gly) and sialylated FN3 (FN3-Sia) produced over 5 h at 28°C in *E. coli* DKK601 cells containing the *FN3* gene alone; *FN3* and *lsgCDEF*, *wecA*, *pglB* and *pglK* genes for *N*-glycosylation (FN3-Gly) (Ding et al., 2017); or additional *neuBCA* and pl-*ST6* genes to effect sialylation (FN3-Sia), detected by **(D)** Coomassie-stained SDS-PAGE, **(E)** Western blotting using anti-FLAG M1 antibody, and **(F)** lectin blotting using Neu5Ac-α-2,6-Gal/GalNAc-specific SNA-I (upper panel) or Gal-β-1,4-GlcNAc-specific ECA (lower panel) lectins. (**D–F**) lane 1–3: purified FN3, FN3-Gly, FN3-Sia. (For detailed information, see Supplementary Figure 5). Data are the means ± standard errors (SD) from three independent representative experiments.

The *E. coli* DH5αΔ*nanKETA*:*kan* (DKK601) strain was generated and utilized to produce terminally sialylated FN3. Growth of the strain in shake flasks was determined to be unaffected by the manipulation compared to the parental *E. coli* DH5α (Supplementary Figure 2). Western blot analysis of initial co-expression of sialylation pathway genes with the FN3 acceptor protein indicated that almost 100% of FN3 molecules exhibited an increase in molecular weight after 3 h induction (Figure 2A, upper panel), while FN3 molecules produced in the presence of pIG6-FN3-Gly-1 alone exhibited no molecular weight shift after up to 5 h induction (Figure 2A, lower panel). The highest yields of putative sialylated FN3 and FN3 were 1.49 ± 0.15 mg/L and 15 ± 0.88 mg/L, respectively, after 4 h induction of IPTG and arabinose (Figure 2B).

To determine the reason for the low level of production of the putatively sialylated FN3, the growth rate of the *E. coli* DKK601/pIG6-Sia + pC15-plsg and DKK601/pIG6-FN3-Gly-1 cells was investigated. Growth rates of the two strains were similar before induction but cells harboring the pIG6-Sia and pC15-plsg plasmids slowed relative to those containing only the pIG6-FN3-Gly-1 plasmid after induction of the full sialylation pathway (Figure 2C), suggesting that a higher metabolic burden was associated with the sialylation procedures.

To confirm the occurrence of sialylation, FN3 produced under varying expression conditions was purified using HisTrap columns and analyzed by Western blotting and lectin blotting using anti-FLAG M1 antibody and Neu5Ac-α-2,6-Gal/GalNAc-specific SNA-I lectin, respectively. Unglycosylated FN3 revealed a single band upon Coomassie Blue staining (Figure 2D) and Western blotting (Figure 2E) but was not detected using either lectin (Figure 2F). Meanwhile, the putatively sialylated FN3 was strongly recognized by sialic acid-binding SNA-I lectin (Figure 2F upper panel), but not detected by Gal-β-1,4-GlcNAc-specific ECA (Figure 2F lower panel), while the glycosylated FN3 containing the tetrasaccharide glycan (Ding et al., 2017) was strongly detected by ECA (Figure 2F lower panel) but not by SNA-I (Figure 2F upper panel). These results indicate the presence of α-2,6-linked sialylation in the glycoengineered FN3. Detection products in lectin blots normally were consistently less intense than in antibody-based Western blots, indicating a possibly lower sensitivity or affinity of the lectin for its glycan target compared to the anti- FLAG M1 antibody.

### LC-MS/MS Analysis of Sialylated Glycoproteins

The sialylated FN3 was further analyzed by intact protein MS. This analysis indicated that 55 ± 5.8% (mean ± SD, *n* = 3) of the putative sialylated FN3 was modified with terminally sialylated glycan (Supplementary Figure 6). As sialic acid moieties in glycans can be dissociated by the ionization process of MALDI, this makes it difficult to correctly determine the concentration of sialylated glycans by MALDI-TOF (Fukuyama et al., 2014). Accordingly, we inferred that the proportion of the sialylated glycoproteins is between the measured values of MS (55%) and lectin (90%) methods.

**FIGURE 3:**
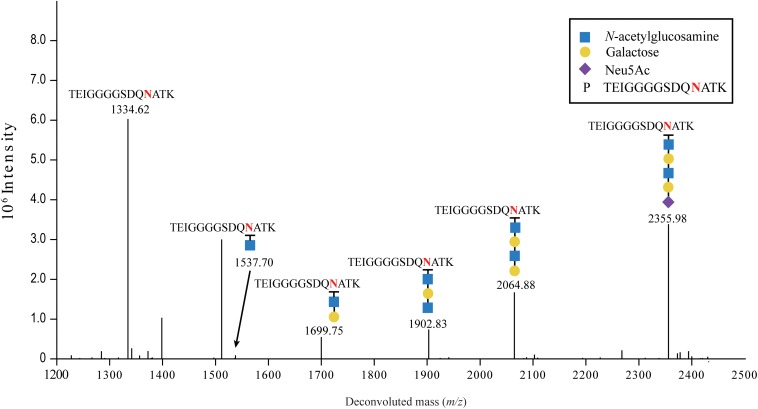
LC-MS/MS analysis of sialylated glycoproteins. Purified FN3 carrying the sialylated glycan was analyzed by LC-MS/MS after enzymatic digestion. The fragmentation spectrum of the Neu5Ac-α-2,6-Gal-β-1,4-GlcNAcβ-1,3-Gal-β-1,3-GlcNAc-TEIGGGGSDQNATK glycopeptide is shown. The peaks at *m/z* 1334.62 and 2355.98 correspond to the TEIGGGGSDQNATK peptide and the sialylated TEIGGGGSDQNATK glycopeptide containing a NeuAc(1)Hex(2)HexNAc(2) glycan, respectively. Peaks at *m/z*, and 2064.88, 1902.83, 1699.75, and 1537.70 correspond to loss of, NeuAc(1), NeuAc(1)Hex, NeuAc(1)Hex(1)HexNAc(1), and NeuAc(1)Hex(2)HexNAc(1), respectively, from the sialylated glycopeptide. Blue squares, yellow circles and purple diamonds indicate GlcNAc, Gal, and Neu5Ac residues, respectively.

Sialylation of FN3 was further confirmed by mass spectrometry after digestion with trypsin and endoproteinase Glu-C, and analysis of the glycan acceptor peptide TEIGGGGSDQ NATK (1104.1 Da) (glycosylated asparagine is underlined) for the presence of covalently attached, terminally sialylated glycans (Figure 3). The parental peak of 2355.98 Da corresponding to the glycan acceptor peptide TEIGGGGSDQNATK carrying NeuAc(1)Hex(2)HexNAc(2) was found in MS/MS spectra (Figure 3), as well as fragmentation peaks of 2064.88 Da, 1902.83 Da, 1699.75 Da, 1537.70 Da, and 1334.62 Da, showing the sequential loss of 291.10 Da (NeuAc), 162.05 Da (Hex), 203.08 Da (HexNAc), 162.05 Da (Hex), and 203.08 Da (HexNAc), respectively. This confirmed that the expected sialylated pentasaccharide, with the sequence NeuAc-Hex-HexNAc-Hex-HexNAc-, was successfully transferred onto the glyco-tagged FN3 acceptor protein.

Taking together the results of Western blotting, lectin blotting and MS/MS spectra, we concluded that terminally sialylated FN3 with an attached Neu5Ac-α-2,6-Gal-β-1,4-GlcNAc-β- 1,3-Gal-β-1,3-GlcNAc glycan was successfully produced in the periplasm of the *E. coli* DKK601 cells.

## Discussion

In this study, we successfully engineered a pathway to produce a terminally sialylated, homogeneous *N*-glycosylated protein with α-2,6-linkages in the periplasm of *E. coli*, and without the requirement to supply sialic acid in the medium. To our knowledge, this is the first demonstration of the production of such a terminally sialylated *N*-glycoprotein, with a sialic acid cap characteristic of human *N*-glycans (Neu5Ac-α-2,6-Gal-β-1,4-GlcNAc-), in *E. coli*. The molecular properties of the sialylated FN3 protein are currently being investigated, including its susceptibility to proteolytic degradation, solubility, persistence in the circulation, and immunogenicity.

Although the sialylated glycoprotein was successfully produced in *E. coli*, induction of the full sialylation pathway resulted in a detrimental effect on host cell growth and a greatly reduced yield of the sialylated protein, from 15 mg/L of unmodified FN3 to a maximum purified yield of 1.5 mg/L of the sialylated FN3. Similar observations have been noted in other studies of recombinant protein glycosylation (Lizak et al., 2011; Hu et al., 2013; Keys et al., 2017) and this effect is likely to be due to the increased metabolic burden associated with expression of the multiple additional genes required for glycosylation, particularly those under the control of strong promoters (Glasscock et al., 2018). Optimization of gene expression levels in the pathway through engineering of promoters and ribosome-binding sites will be important to better balance the protein expression, LLOs production and glycosylation processes, thereby reducing the metabolic burden. Plasmid loss has also been identified as a basis for reduced yields (Lizak et al., 2011; Hu et al., 2013), with host cells required to maintain two or three compatible plasmids with different selectable markers to accommodate the multiple genes necessary for protein expression and glyco-modification. Previous results from our group indicated that FN3 could be produced in considerably higher amounts in *E. coli* CLM37 (Ding et al., 2017) cells than in the present *E. coli* DKK601 cells and so further strain screening will be carried out to increase yields of the sialylated *N*-glycoprotein. Future work will focus on strain screening to improve stability during protein expression, and combining glycoengineering genes onto a single plasmid or into the bacterial genome to increase sialylated protein yields (Strutton et al., 2018; Yates et al., 2019). Evaluation of the sialylation efficiencies of other well characterized bacterial α-2,6-STases is also underway in our laboratory to further increase sialylation efficiency and build on the present breakthrough. The strategy reported in this study will be valuable for constructing *E. coli* strains capable of producing high yields of terminally sialylated *N*-glycoproteins.

## Conclusion

The work presents the first pathway for the production of terminally sialylated, α-2,6-linked *N*-glycoproteins in *E. coli*. As the engineered *E. coli* strain also synthesizes the CMP-Neu5Ac required for sialylation, this further eliminates the requirement to supply exogenous Neu5Ac in the medium and renders the process amenable to scale-up. The work constitutes an important step in efforts toward creating human-like recombinant glycoproteins in *E. coli.*

## Data Availability Statement

The raw data supporting the conclusion of this manuscript will be made available by the authors, without undue reservation, to any qualified researcher.

## Author Contributions

XH and ND conceived the project. JZ, YR, XF, LZ, GG, TZ, and YZ performed the experiments and analysis the data. JW and ND analyzed the data and revised the manuscript. XH, ND, and JZ wrote the manuscript. All authors contributed to manuscript revision, and read and approved the submitted version.

## Conflict of Interest

The authors declare that the research was conducted in the absence of any commercial or financial relationships that could be construed as a potential conflict of interest. A patent application has been filed by Dalian University.
