# Supplementary material for: An Engineered Pathway for Production of Terminally Sialylated N-glycoproteins in the Periplasm of Escherichia coli
Source: Front Bioeng Biotechnol. 2020 Apr 15;8:313. doi: 10.3389/fbioe.2020.00313 (PMC7174548; doi:10.3389/fbioe.2020.00313)
Supplement: Supplementary file 1 [file Data_Sheet_1.docx]

Supplementary Material

# Supplementary Method

**1.1 Construc****tion of *E. coli* DH5αΔ*nanKETA*::*kan*Strain**

*E. coli* DH5αΔ*nanKETA*::*kan* strain was constructed by knocking out the *nanKETA* genes in *E. coli* DH5α. The *nanKETA*gene locus was disrupted by removing a3335 bp segment from the chromosomal DNA using the Red homologous recombination systemas previously described (Datsenkoet al., 2000; Fierfort et al., 2008). The additional plasmids and the upstream and downstream deletion primers used in this study are listed in **Supplementary Table1**. Primers JD*nanKETA*R and JD*nanKETA*F were used to identify mutants. Primers used are listed in **Supplementary Table1**.

**1.2Analysis ofSialylated FN3via MALDI-TOF Detection**

The relative intensity of sialylated FN3or un-sialylatedFN3-Glywascalculatedusing area under the curve (AUC) analysis inGraphpad Prism 5.5. The AUC of individual peaks of MALDI-TOF chromatography were calculated using triplicate data, yieldingone value each for the area under the un-sialylatedFN3 curve and thesialylatedFN3 peaks.Peak area ratios of sialylatedFN3-Sia/un-sialylatedFN3-Glywere analyzedfrom three independent MALDI-TOF spectra, revealing 55 ± 5.8% (mean ± SD, n=3) of the FN3 protein was modified with sialylated glycan.

# Supplementary Figures and Tables

## Supplementary Figures

**AB**

**
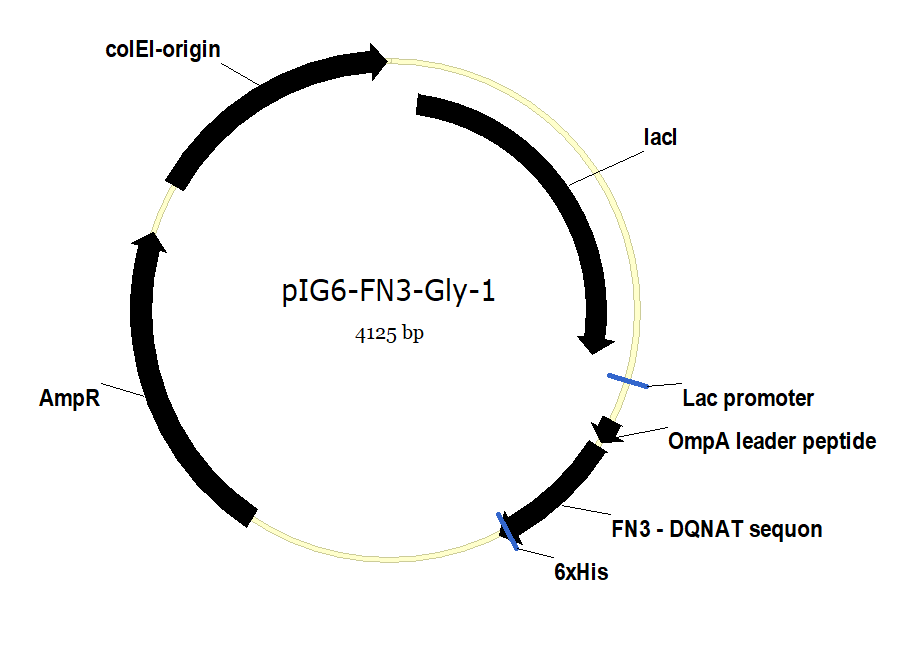
**
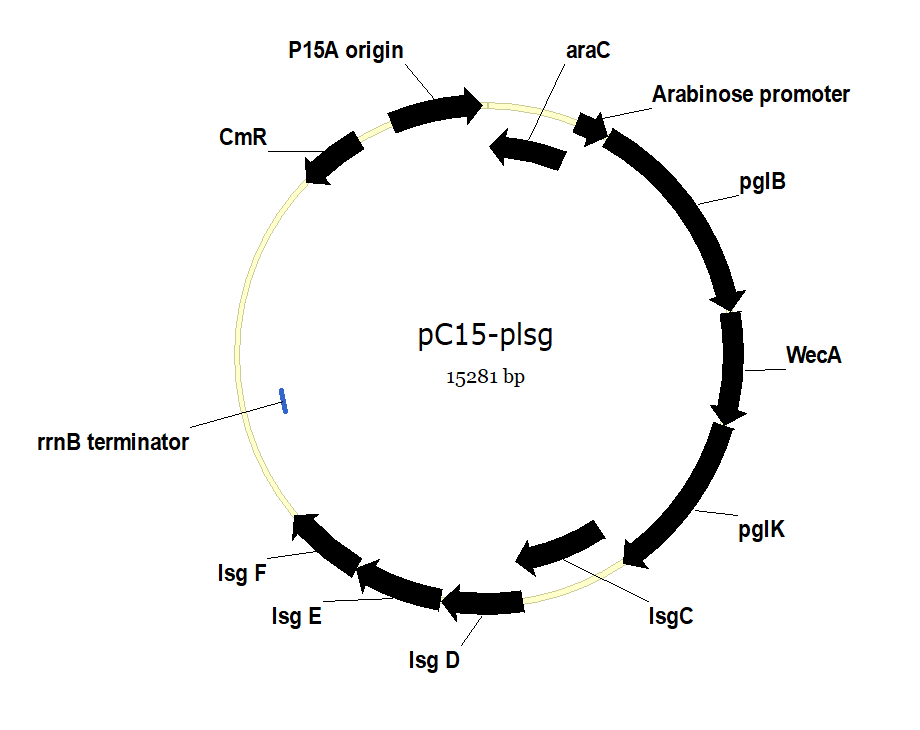


**C**


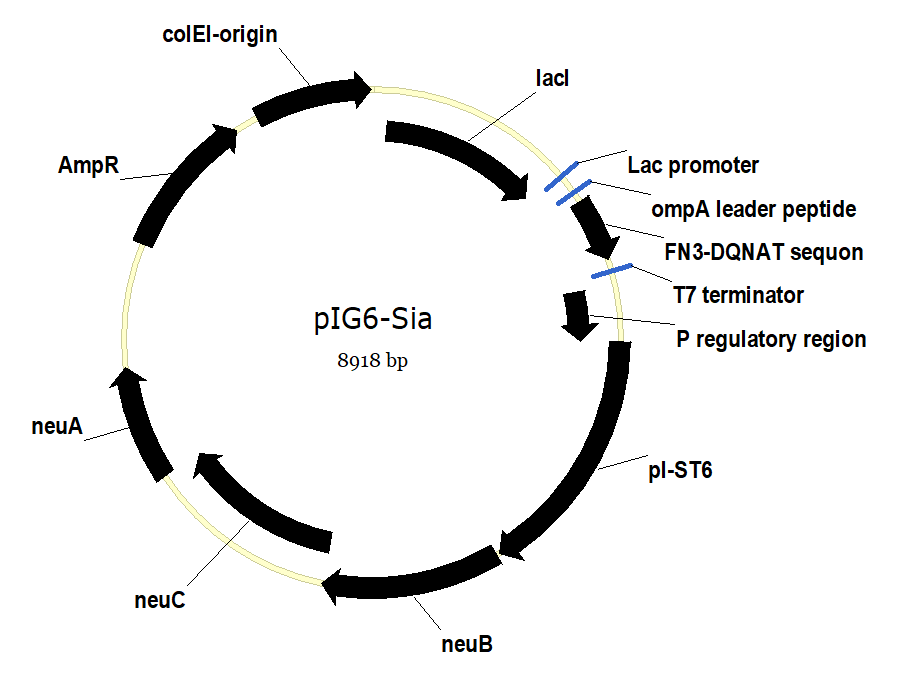


**Supplementary Figure 1.** Plasmid maps. **(A)** pIG6-FN3-Gly containing the FN3 gene in an *ompA* leader-FLAG-FN3-DQNAT glycotag-6xHis format under expression control of a*Lac* promoter. **(B)** pC15-plsg containing a cluster of*pglB*, *wecA*, *pglK* and *lsgCDEF* genes under the control of an arabinose promoter. **(C)** pIG6-Sia plasmid containing the FN3 gene in an *ompA* leader-FLAG-FN3-DQNAT glycotag-6xHis format under the control of a *lac* promoter, and a gene cluster including *P. leiognathi* α-2,6-STase *pl-ST6* and *neuBCA* genes under the control of a weak constitutivepromoter (P) from the *N*-glycosylation*pgl* locus of*Campylobacter jejuni*.


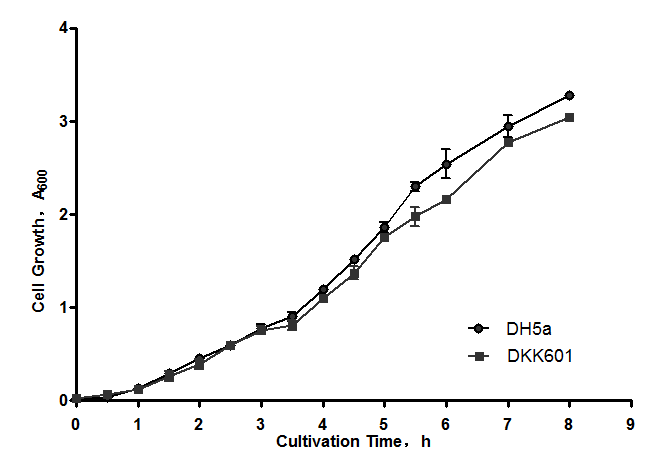


**Supplementary Figure 2.** Growth curves of *E. coli* DH5α and DKK601 (DH5αΔ*nanKETA*::*kan*) strains under non-induced conditions. The growth patterns of both strains were similar during the 8 h cultivation. Data were analyzed on GraphPad Prism 5.5 and are presented as mean values of three measurements, with error bars indicating the standard deviation.


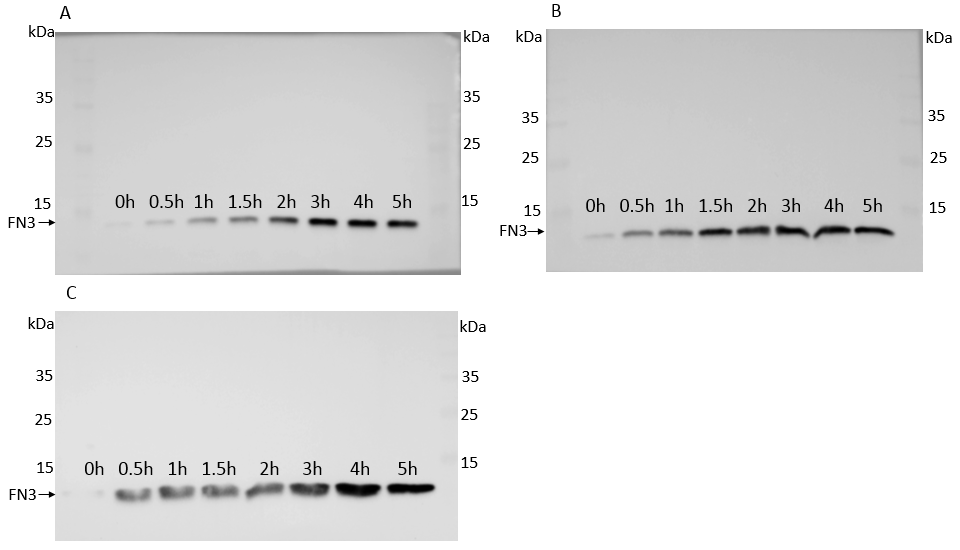


**Supplementary Figure 3.** Westernblot analysis of the expression of unmodified FN3. The cell lysates at different time points were collected from *E. coli* DKK601/ pIG6-FN3-Gly-1 cells and detected using an anti-FLAG M1 antibody.The highest expression level of FN3 was determined by comparison topurified FN3.


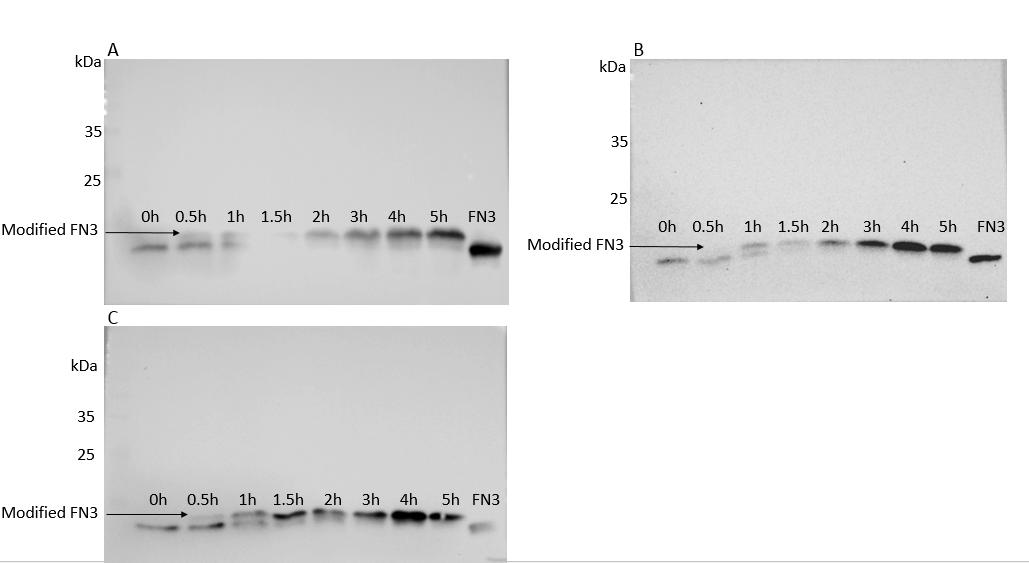


**Supplementary Figure 4.** Western blot analysis of expression of modified FN3. Cell lysates were prepared from *E. coli* DKK601/ pIG6-Sia+pC15-plsg cultures at the indicated time points, followed by detectionusing an anti-FLAG M1 antibody. Purified FN3 (farthest most right lane in each gel) was used as a standard for quantification.Samples were analyzed using GraphPad Prism 5.5 and presented as mean values of three measurements, with error bars indicating the standard deviation.


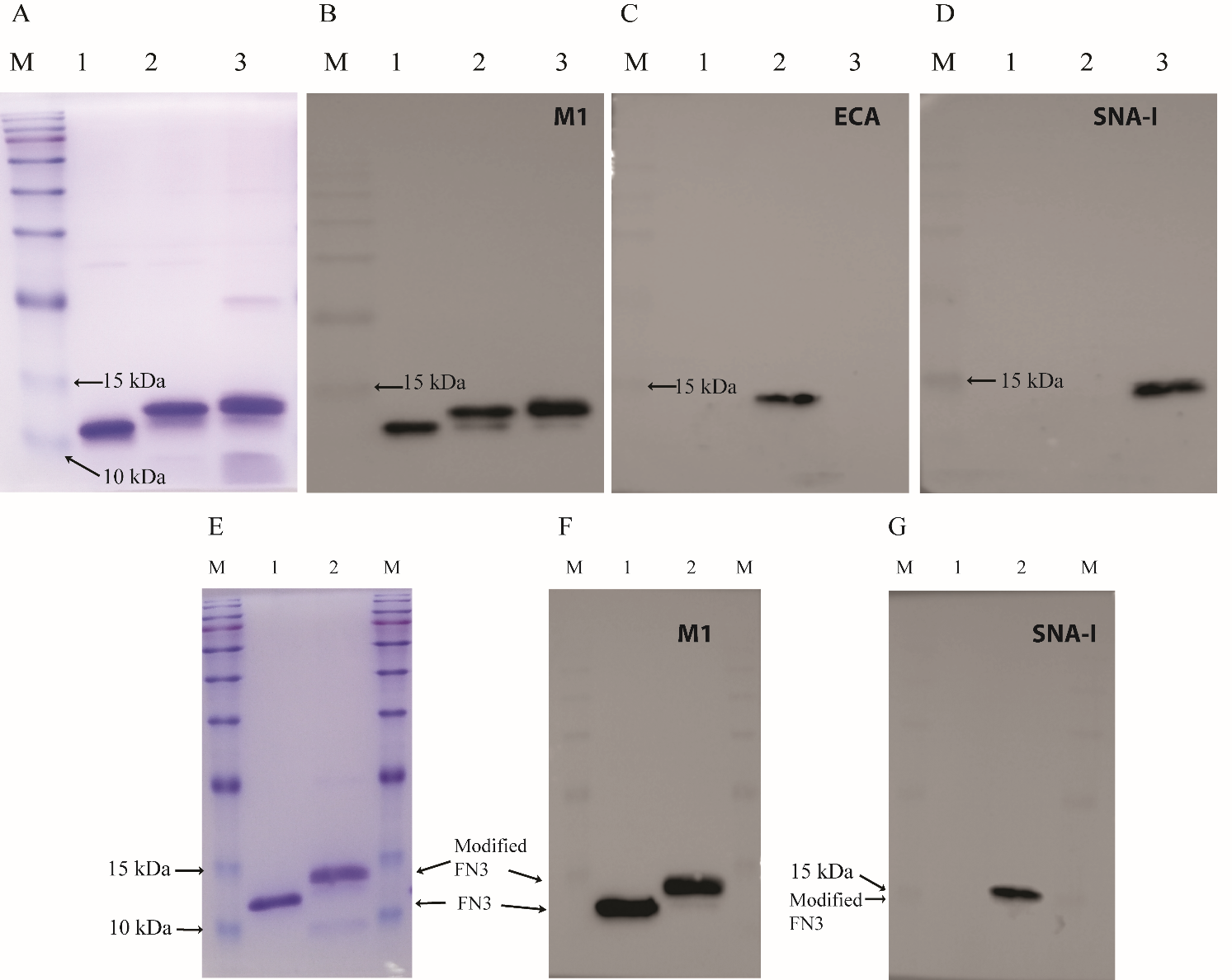


**Supplementary Figure 5.**Purified unmodified FN3, *N*-glycosylated FN3-Glyand sialylated FN3-Sia produced over 5 h at 28 °C in *E. coli* DKK601 cells containing the *FN3* gene alone (FN3); *FN3*-*Gly* and *lsgCDEF*, *wecA*, *pglB* and *pglK* genes for N-glycosylation (FN3-Gly) (Ding et al., 2017); or additional *neuBCA* and *pl*-*ST6* genes to effect sialylation (FN3-Sia), detected by **(A)** Coomassie-stained SDS-PAGE, **(B)** Western blotting using anti-FLAG M1 antibody and **(C)** lectin blotting using Gal-β-1,4-GlcNAc-specific ECA or**(D)** Neu5Ac-α-2,6-Gal/GalNAc-specific SNA-I lectin; detected **(E)**Coomassie-stained SDS-PAGE, **(F)** Western blotting using anti-FLAG M1 antibody, **(G)** Neu5Ac-α-2,6-Gal/GalNAc-specific SNA-I lectin. (**A-D**) lane 1-3: purified FN3, FN3-Gly, FN3-Sia; (**E-G**) lane 1-2: purified FN3, FN3-Sia.


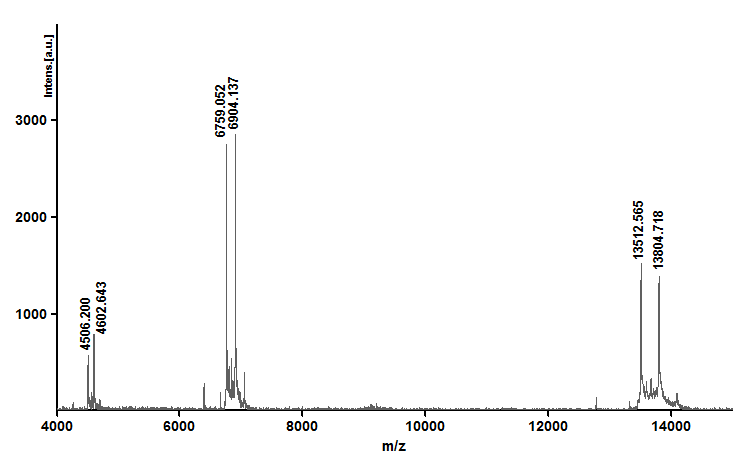


**Supplementary Figure 6.**MALDI-TOF analysis of purified *N*-linked modifiedFN3.Total glycoprotein products from three biological replicateswere affinity purified and analyzed by MALDI-TOF. The represented graph mainly depicts the relative intensity of sialylated modified FN3and incompletely modified FN3-Sia. With different levels of ionized energy, the peaks at *m/z*4560.200, 6759.052, 13512.565 correspond to FN3 modified with the Hex(2)HexNAc(2) glycan. The peaks at *m/z*4602.643, 6904.137 and 13804.718 correspond to theFN3 carrying NeuAc-Hex(2)HexNAc(2) glycan. Quantification of each species is based on peak areas using AUC (area under the curve) analysis of GraphPad Prism 5.5. Theaverage percentage(means ±SD, n=3) was calculated from triplicate data. Full spectra are provided in the Source data file associated with this manuscript.

**Supplementary Tables**

**Supplementary Table 1.** Primers used in this study

| Primers for deletion | Sequences(5'−3') | |
| --- | --- | --- |
| Del *nanKETA* R1  Del *nanKETA* F1  Del *nanKETA*R2  Del *nanKETA* F2 | | tggtgtacaacattccagccctgagtggggtaaaactctgtcaaacatgagaattaa  gcatccgcgccagccaactccccctgcgctgccgctgcgtgtaggctggagctgcttgcaattattgattcggcgg  atggtttgccgatggtggtgtacaacattccagccctgag  gtcaccctgcccggcgcgcgtgaaaatagttttcgcatccgcgccagccaactccccct |
| Primers for identification | |  |
| JD *nanKETA* R  JD*nanKETA* F | | cgcactggcaatcagttgtg  cgtcacgccgttctactatc |

**REFERENCES**

Datsenko, K. A. and B. L. Wanner (2000). One-step inactivation of chromosomal genes in *Escherichia coli* K-12 using PCR products. *Proc Natl AcadSci U S A*. 97(12): 6640-5.doi: 10.1073/pnas.120163297.

Fierfort, N., Samain, E., (2008). Genetic engineering of *Escherichia coli* for the economical production of sialylated oligosaccharides. *J. Biotechnol*. 134, 261-5.doi: 10.1016/j.jbiotec.2008.02.010.
